# Epidemiological Differences in the Impact of COVID-19 Vaccination in the United States and China

**DOI:** 10.3390/vaccines9030223

**Published:** 2021-03-05

**Authors:** Monia Makhoul, Hiam Chemaitelly, Houssein H. Ayoub, Shaheen Seedat, Laith J. Abu-Raddad

**Affiliations:** 1 Infectious Disease Epidemiology Group, Weill Cornell Medicine-Qatar, Cornell University, Qatar Foundation-Education City, Doha 24144, Qatar; mom2039@qatar-med.cornell.edu (M.M.); hsc2001@qatar-med.cornell.edu (H.C.); shs4004@qatar-med.cornell.edu (S.S.); 2 World Health Organization Collaborating Centre for Disease Epidemiology Analytics on HIV/AIDS, Sexually Transmitted Infections, and Viral Hepatitis, Weill Cornell Medicine–Qatar, Cornell University, Qatar Foundation–Education City, Doha 24144, Qatar; 3 Department of Population Health Sciences, Weill Cornell Medicine, Cornell University, New York, NY 10022, USA; 4 Department of Mathematics, Statistics, and Physics, Qatar University, Doha 2713, Qatar; hayoub@qu.edu.qa

**Keywords:** severe acute respiratory syndrome coronavirus 2 (SARS-CoV-2), COVID-19, coronavirus, epidemiology, vaccine, mathematical model

## Abstract

This study forecasts Coronavirus Disease 2019 (COVID-19) vaccination impact in two countries at different epidemic phases, the United States (US) and China. We assessed the impact of both a vaccine that prevents infection (*VE*_S_ of 95%) and a vaccine that prevents only disease (*VE*_P_ of 95%) through mathematical modeling. For *VE*_S_ of 95% and gradual easing of restrictions, vaccination in the US reduced the peak incidence of infection, disease, and death by >55% and cumulative incidence by >32% and in China by >77% and >65%, respectively. Nearly three vaccinations were needed to avert one infection in the US, but only one was needed in China. For *VE*_P_ of 95%, vaccination benefits were half those for *VE*_S_ of 95%. In both countries, impact of vaccination was substantially enhanced with rapid scale-up, vaccine coverage >50%, and slower or no easing of restrictions, particularly in the US. COVID-19 vaccination can flatten, delay, and/or prevent future epidemic waves. However, vaccine impact is destined to be heterogeneous across countries because of an underlying “epidemiologic inequity” that reduces benefits for countries already at high incidence, such as the US. Despite 95% efficacy, actual vaccine impact could be meager in such countries if vaccine scale-up is slow, acceptance is poor, or restrictions are eased prematurely.

## 1. Introduction

With over 80 million infections and a death toll approaching two million [1], the severe acute respiratory syndrome coronavirus 2 (SARS-CoV-2) pandemic has been one of the most challenging global health emergencies in recent history [2]. The unparalleled burden on healthcare systems has necessitated unprecedented restrictions on mobility and on social and economic activities [3,4]. The ensuing losses to national and global economies are probably the largest since the Great Depression [2].

We previously developed a mathematical model to investigate the generic population-level impact of SARS-CoV-2 vaccination [5]. In light of recently produced vaccines with ~95% efficacy against Coronavirus Disease 2019 (COVID-19) symptomatic disease [6,7], the model was extended to assess the impact of these novel vaccines on COVID-19 morbidity and mortality in two major countries at different epidemic phases, the United States (US) and China. The impact was assessed under two different assumptions for the mechanism of action of the vaccine—that it prevents both infection and disease or that it prevents only disease. The impact was further assessed at different levels of vaccine coverage, different time courses for vaccine scale-up, and different schedules for easing of social and physical distancing restrictions following the launch of vaccination. 

## 2. Materials and Methods

### 2.1. Mathematical Model and Parameterization

The extended model was age-structured, stratifying the population into cohorts based on vaccination status, age group, infection status, infection stage, and disease stage. Vaccination was defined as completion of two vaccine doses [6,7,8]. Population movement among cohorts was determined using a set of coupled nonlinear differential equations. Given interest in assessing vaccination impact in the short-term (over only 2021), demography was assumed stable. Contact between individuals in different age groups was determined using an age-mixing matrix that allowed a range of assortativeness in mixing. Details of the model are in Supplementary Information Text S1A, S1B and Figures S1 and S2. The model was coded, fitted, and analyzed using MATLAB R2019a [9].

Since the evidence suggests that reinfection with this virus is a rare event [10,11,12,13,14,15], those recovered from infection were assumed protected against reinfection, but only for one year, based on the behavior of other “common cold” coronaviruses [16]. For the same purpose, it was assumed that vaccine-induced immunity will also last for only one year, a conservative assumption given that studies have been increasingly suggesting that immunity may be more durable and long-lasting [10,15,17,18,19]. The waning of both natural and vaccine immunity was assumed to follow a gamma distribution of order n=15. That is, most people lose their immunity after about one year, and only a small minority lose their immunity after a period that is either much shorter or much longer than one year (Figure S3). 

The model was parameterized using state-of-the-art empirical evidence for the infection’s natural history and epidemiology. The natural history and the distribution of infected individuals across mild (or asymptomatic), severe, or critical infection stages and the infection mortality rate in each age group were based on natural history studies [20,21,22,23,24,25,26,27] and existing comprehensive analyses of analyzed epidemics [16,28,29] that followed the World Health Organization (WHO) guidelines for classifying infection severity [30] and determining COVID-related death [31]. All age groups were assumed (biologically) equally susceptible to this infection. Population demographic information (size, age distribution, and life expectancy) were obtained from the United Nations World Population Prospects database [32]. Details of model parameters, values, and justifications are in Supplementary Information Text S1C and Tables S1 and S2.

### 2.2. Characteristics of the Vaccine and Scale-Up Scenarios

Since the primary endpoint of the vaccine’s randomized clinical trials was efficacy of the vaccine against laboratory-confirmed COVID-19 cases [6,7,8], and not just any infection, documented or undocumented, it is unknown whether the vaccine prophylactically reduces susceptibility to the infection (that is, $VE_{S}$ efficacy defined as the proportional reduction in the susceptibility to infection among those vaccinated compared to those unvaccinated [5]) or whether it just reduced serious symptomatic COVID-19 cases with no effect on infection (that is, $VE_{P}$ efficacy against disease progression, defined as the proportional reduction in the fraction of individuals with severe or critical infection among those vaccinated but who still acquired the infection compared to those unvaccinated [5]). These two mechanisms of action bracket the two extremes for the vaccine’s biological effect, with the former mechanism being the most optimistic (reducing both infection and disease) and the latter being the most pessimistic (reducing only disease).

Notwithstanding this uncertainty, the impact of the vaccine was assessed under each of these mechanisms of action, assuming $VE_{S}=95\%$ or $VE_{P}=95\%$, thus covering the spectrum for potential vaccine impact. However, further more specific estimates can be generated as data on the vaccines’ mechanisms of action become available. In the baseline scenario, the vaccine was introduced in both countries on 1 January 2021 with a scale-up to reach vaccine coverage of 80% by the end of 2021. Given that the purpose of vaccination is to alleviate the need for restrictions that have stifled social and economic activities, social distancing restrictions were assumed to be eased gradually over six months, so that “normalcy” would be attained at the end of these six months. Normalcy was defined as a social contact rate in the population equal to that prior to the pandemic.

Since the US has experienced a large epidemic, it was assumed that 20% of the US population has already been infected by 1 January 2021 [33], with those already infected (if subsequently vaccinated) assumed to have natural immunity against reinfection as supported by reinfection studies [10,15,19]. Moreover, the basic reproduction number at time of onset of vaccination was assumed at $R_{0}=1.2$, reflecting the current phase of an expanding epidemic. It was also assumed that $R_{0}$ will gradually increase with easing of restrictions to reach $R_{0}=4$ at the end of six months. The value of $R_{0}=4$ is justified by existing estimates assuming a “natural” epidemic in the absence of non-pharmaceutical interventions and by evidence of higher infectiousness for this virus after the emergence of the D614G mutation [34,35,36,37] and of new variants for this virus [38]. 

For China, it was assumed that the entire population is still susceptible to the infection, given the small number of documented infections relative to its large population size, and that the epidemic was contained [1,25]. Moreover, it was assumed that $R_{0}=1$ at the onset of vaccination (reflecting the non-expanding epidemic), but that $R_{0}$ will gradually increase with easing of restrictions to reach an $R_{0}=4$ at the end of six months. 

### 2.3. Measures of Vaccine Impact

The population-level impact of SARS-CoV-2 vaccination was assessed by quantifying incidence, cumulative incidence, and reduction in incidence of infections, severe disease cases, critical disease cases, and COVID-19 deaths arising in the presence of vaccination compared to the counter-factual scenario of no vaccination. These measures of vaccine impact factor both direct and indirect public health benefits of vaccination, that is, the direct effects of the vaccine due to $VE_{S}$ or $VE_{P}$ efficacies and the indirect effects due to the reduction in onward infection transmission given higher proportions of immune individuals (applicable only in the case of $VE_{S}$). Vaccine effectiveness, that is, number of vaccinations needed to avert one infection or one adverse disease outcome (ratio of the number of vaccinations relative to the number of averted outcomes), was further calculated using the different scenarios to inform future cost-effectiveness analyses. In this regard, vaccine “effectiveness” is not to be confused with vaccine biological “efficacy”, which is assumed constant at 95% [6,7]. Impact of vaccination prioritization by age was not assessed, as it was investigated in earlier studies [5,39].

### 2.4. Uncertainty Analysis

A multivariable uncertainty analysis was conducted to determine the range of uncertainty for model predictions using 500 model runs. At each run, Latin Hypercube sampling [40,41] was applied to select vaccine efficacy from within its reported credible range [7] and to select a vaccine duration of protection within ±30% of one year duration. The resulting distribution for vaccine impact across all 500 runs was used to calculate the predicted means of different outcomes and the uncertainty associated with those means.

## 3. Results

**Figure 1:**
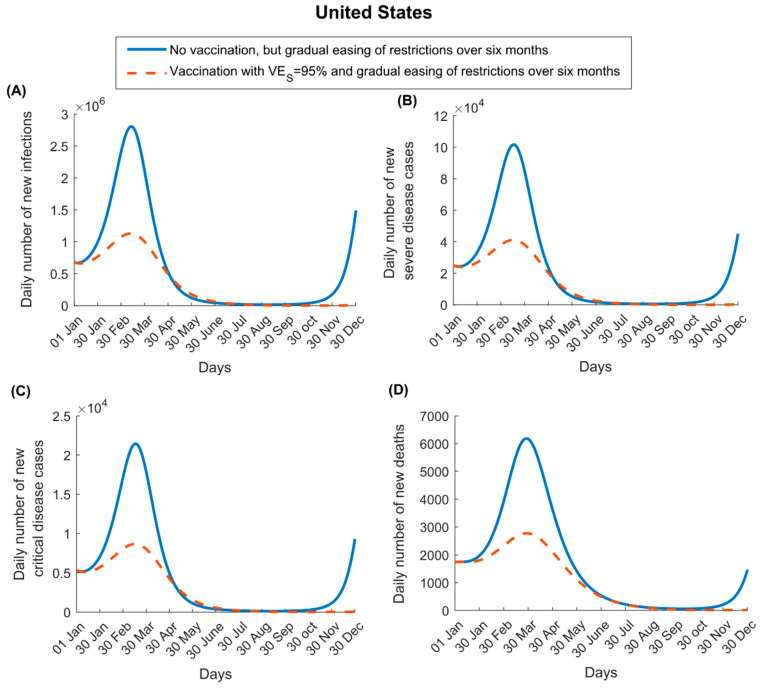
Impact of severe acute respiratory syndrome coronavirus 2 (SARS-CoV-2) vaccination on numbers of (**A**) new infections, (**B**) new severe disease cases, (**C**) new critical disease cases, and (**D**) new deaths in the United States. The vaccine is assumed to have an efficacy of 95% against infection and is introduced on 1 January 2021, when the proportion of the population already infected is 20%. Vaccine coverage is scaled up to reach 80% by 31 December 2021 (Figure S4A shows the vaccine coverage scale-up in the United States over time). Duration of both vaccine protection and natural immunity is one year. This scenario assumes an $R_{0}$ of 1.2 on 1 January 2021, which increases with gradual easing of restrictions to reach 4.0 after six months. Results of a scenario assuming that the vaccine has no efficacy against infection but an efficacy of 95% against severe and critical disease are shown in Figure S5.

For $VE_{S}=95\%$, vaccination in the US flattened the epidemic curve but did not prevent a new epidemic wave, though it resulted in a smaller one, with the assumed gradual easing of restrictions following the onset of vaccination (Figure 1). The vaccine reduced peak incidence of infection, severe disease, critical disease, and COVID-19 death by 59.6%, 59.5%, 59.0%, and 55.3%, respectively, and cumulative number of infections, severe disease cases, critical disease cases, and deaths by 35.7%, 35.2%, 35.0%, and 32.7%, respectively, by the end of 2021. However, incidence started to increase toward the end of 2021, as vaccine immunity waned and those previously infected began losing their protective immunity against reinfection. 

For $VE_{P}=95\%$, the vaccination had no impact on infection (as it does not protect against infection) and less impact on disease and death (Figure S5). Peak incidence of severe disease, critical disease, and death was reduced by only 22.0%, 22.0%, and 21.1%, respectively. The cumulative numbers of severe disease cases, critical disease cases, and deaths were reduced by only 17.4%, 17.2%, and 16.7%, respectively, by the end of 2021. 

**Figure 2:**
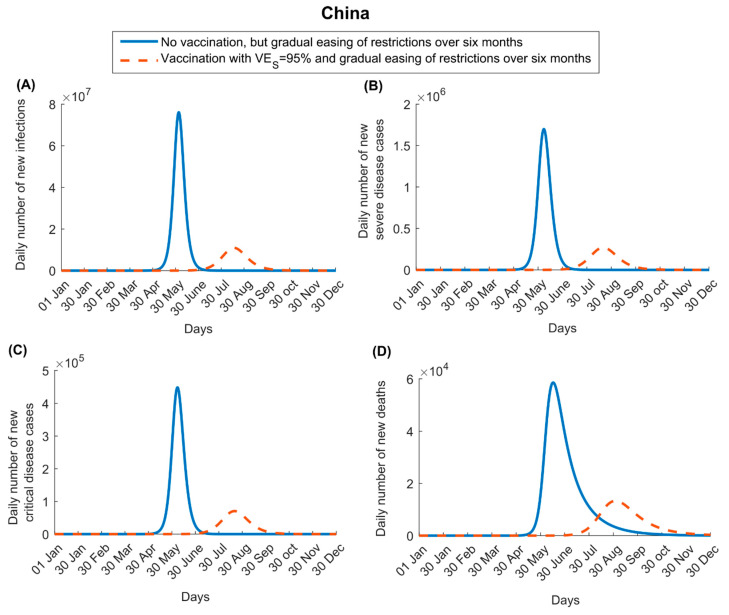
Impact of SARS-CoV-2 vaccination on numbers of (**A**) new infections, (**B**) new severe disease cases, (**C**) new critical disease cases, and (**D**) new deaths in China. The vaccine is assumed to have an efficacy of 95% against infection and is introduced on 1 January 2021. Vaccine coverage is scaled up to reach 80% by 31 December 2021 (Figure S4B shows the vaccine coverage scale-up in China over time). Duration of both vaccine protection and natural immunity is one year. This scenario assumes an $R_{0}$ of 1.0 on 1 January 2021, which increases with gradual easing of restrictions to reach 4.0 after six months. Results of a scenario assuming that the vaccine has no efficacy against infection but an efficacy of 95% against severe and critical disease are shown in Figure S6.

In China, the impact of vaccination was larger than in the US, as the vaccine was introduced at a time when disease incidence was negligible. For $VE_{S}=95\%$, vaccination not only flattened the epidemic curve but also delayed it by a few months (Figure 2). The vaccine reduced peak incidence of infection, severe disease, critical disease, and death by 85.6%, 84.2%, 84.3%, and 77.3%, respectively, and cumulative number of infections, severe disease cases, critical disease cases, and deaths by 65.7%, 65.0%, 65.3%, and 65.3%, respectively, by the end of 2021. Unlike the US, no epidemic re-emergence is seen in China towards the end of the year 2021, as the vast majority of infections in this country would have happened during the course of that year, and thus recovered persons would not have lost their immunity by the end of that year.

For $VE_{P}=95\%$, the vaccination had less impact on disease and death (Figure S6). Peak incidence of severe disease, critical disease, and death was reduced by 44.5% for all of these indicators as well as for their cumulative numbers.

**Figure 3:**
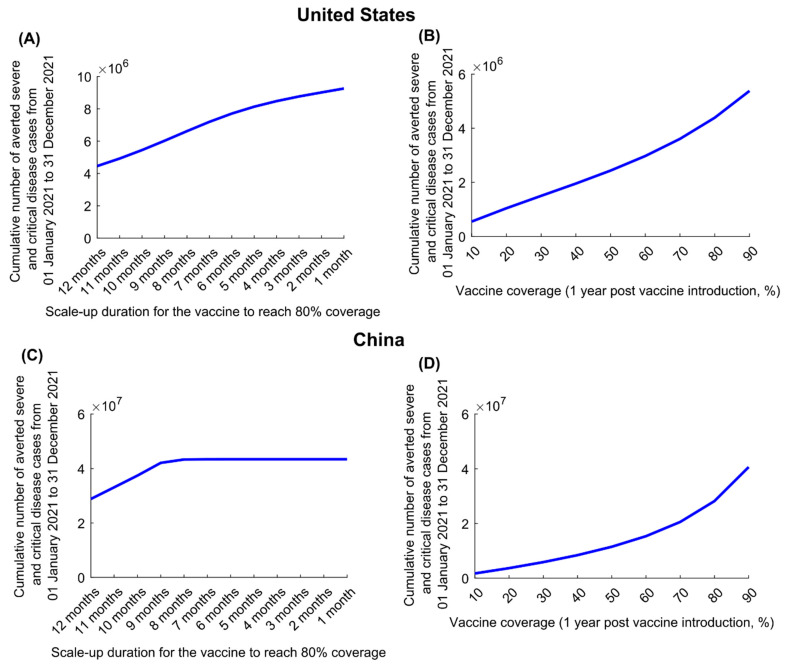
Impact of vaccine scale-up duration and vaccine coverage on numbers of averted severe and critical disease cases for a vaccine that protects against both infection and disease. Cumulative averted severe and critical disease cases in (**A**) the United States and (**C**) China at different vaccination scale-up durations to reach 80% coverage. Cumulative averted severe and critical disease cases in (**B**) the United States and (**D**) China at varying levels of vaccine coverage. The vaccine is assumed to have an efficacy of 95% against infection and is introduced on 1 January 2021, when the cumulative proportion of the population infected is 20% in the United States and 0% in China. Duration of both vaccine protection and natural immunity is one year. This scenario assumes gradual easing of restrictions within 6 months. The results of a scenario assuming the vaccine has no efficacy against infection but an efficacy of 95% against severe and critical disease is shown in Figure S7.

In the US, for $VE_{S}=95\%$, the cumulative number of averted disease cases increased steadily in response to shorter scale-up (to 80% coverage) (Figure 3A). However, in China, there was no additional benefit to be had by shortening scale-up to less than 8 months, as the epidemic was fully contained (Figure 3C). Similar results were obtained for $VE_{P}=95\%$, as shown for the US and China (Figure S7A,C), respectively.

For $VE_{S}=95\%$, the cumulative number of averted disease cases increased steadily with higher vaccine coverage (by end of 2021) in both countries (Figure 3B,D). The gains in averted disease cases increased sharply as vaccine coverage exceeded 70% in the US and 50% in China, because such coverage prevented a much larger epidemic wave. Similar results were obtained for $VE_{P}=95\%$ in both the US and China (Figure S7B,D, respectively).

**Figure 4:**
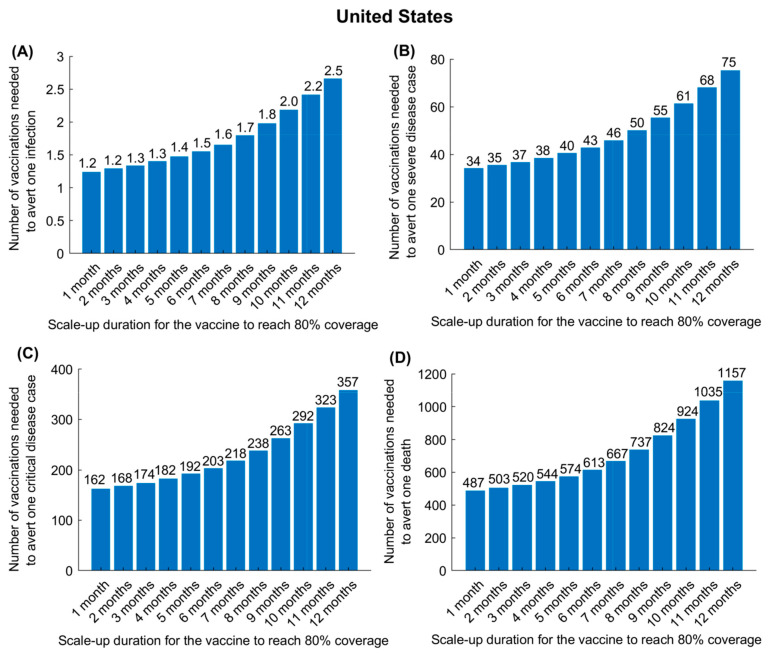
Impact of vaccine scale-up duration on the number of vaccinations needed to avert one infection (**A**), one severe disease case (**B**), one critical disease case (**C**), and one death (**D**) in the United States. The vaccine is assumed to have an efficacy of 95% against infection and is introduced on 1 January 2021, when the cumulative proportion of the population infected is 20%. Duration of both vaccine protection and natural immunity is one year. This scenario assumes a gradual easing of restrictions within 6 months. Corresponding results for China are shown in Figure S8.

In the US, for $VE_{S}=95\%$, the effectiveness of the vaccine in preventing infection (Figure 4A), severe disease (Figure 4B), critical disease (Figure 4C), and death (Figure 4D) was substantially enhanced by more rapid scale-up to reach 80% coverage, since the epidemic was already at high incidence at time vaccination was launched. Whereas, in the US, only one vaccination was needed to avert one infection, provided that scale-up could be accomplished in 6 months, nearly three vaccinations were needed to avert one infection if the scale-up required 12 months. This, however, was not the case in China (Figure S8). Regardless of the speed of scale-up, only one vaccination was needed to avert one infection. 

**Figure 5:**
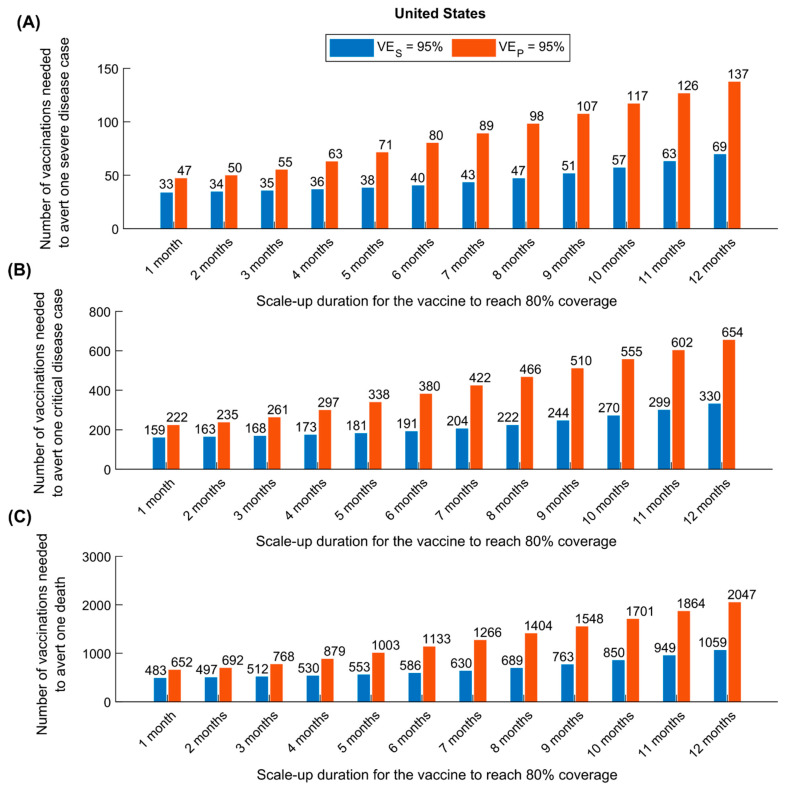
Comparison of the impact of a vaccine acting against infection (*VE_S_* efficacy) versus a vaccine acting only against disease (*VE_P_* efficacy) in the United States. The number of vaccinations needed to avert one severe disease case (**A**), one critical disease case (**B**), and one death (**C**) for a vaccine with $VE_{S}=95\%$ versus a vaccine with $VE_{P}=95\%$. The vaccine is introduced on 1 January 2021, when the cumulative proportion of the population infected is 20%. Duration of both vaccine protection and natural immunity is one year. This scenario assumes a gradual easing of restrictions within 6 months. Corresponding results for China are shown in Figure S9.

Figure 5 shows a comparison of the impact of vaccine scale-up duration on the number of vaccinations needed to avert one severe disease case (Figure 5A), one critical disease case (Figure 5B), and one death (Figure 5C), in the US, between the assumption of $VE_{S}=95\%$ and that of $VE_{P}=95\%$. As expected, a vaccine that prevents infection (and consequently disease) was superior to a vaccine preventing only disease. That superiority was even greater if scale-up is longer, where twice as many vaccinations were needed to avert each of these outcomes. Similar results were obtained for China (Figure S9). 

In all of the above scenarios, it was assumed that easing of social restrictions would occur during six months following initiation of vaccination. However, as expected, a longer duration for easing restrictions resulted in a more favorable impact of vaccination in both the US and China (Figure S10). 

**Figure 6:**
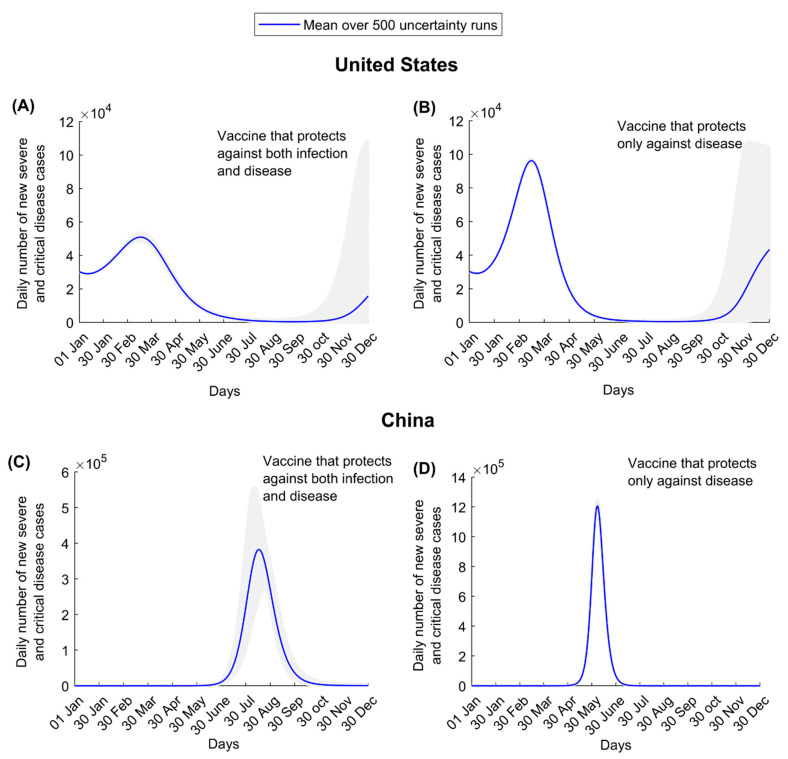
Numbers of new severe and critical disease cases in the United States assuming (**A**) a vaccine that protects against both infection and disease and (**B**) a vaccine that protects only against disease. Numbers of new severe and critical disease cases in China assuming (**C**) a vaccine that protects against both infection and disease and (**D**) a vaccine that protects only against disease. These scenarios assume gradual easing of restrictions within 6 months. Shaded areas are the results of the 500 uncertainty runs, while the solid line is the mean of those runs.

Uncertainty regarding the projected impact was small in the short-term for the US and China in the first wave after vaccinations commenced, but it was large toward the end of 2021, as expected, due to uncertainty about persistence of the vaccine’s protective immunity (Figure 6). 

## 4. Discussion

The key conceptual finding that emerges from this study is that vaccine impact is strongly dependent on the difference between two essential metrics, “time to infection” and “time to vaccination”. The competing “hazard” dynamics between the event of infection and the event of vaccination explain the variability of impact under the parameters considered: incidence at the onset of vaccination, duration of scale-up, vaccine coverage, or timing of the easing of restrictions. As the average time to vaccination is shortened relative to the average time to infection, by altering these parameters (by more rapid scale-up, slower easing of restrictions, or reducing infection incidence through lockdowns or other restrictive measures), the impact would be more favorable, and fewer vaccinations would be needed to avert one infection or disease outcome. 

A striking demonstration of this concept’s importance can be seen in the case of a vaccine that does not prevent infection but prevents disease with $VE_{P}=95\%$ (Figures S4 and S5). This vaccine would not affect the time to infection in the population, and any easing of restrictions with vaccination will shorten the time to infection. Accordingly, such a vaccine, despite its 95% efficacy, would end up averting <20% of disease cases and deaths in the US and <50% in China. Since the time to infection in the US is much shorter than in China, as a consequence of the current high incidence rate, vaccine impact will be more favorable in China, where vaccination can be scaled up over a longer duration and still have superior impact to that in the US. 

A consequence of the above findings is that vaccine impact will likely be heterogenous among nations. Countries with low or negligible incidence will benefit most from vaccination. Vaccine cost-effectiveness will be also optimized in such countries, with only one vaccination needed to avert one infection for a vaccine with $VE_{S}=95\%$ (Figure S8).

Several other findings emerged from this study. Vaccination will flatten the epidemic curve but may not prevent (or delay) a new wave, unless it is scaled up very rapidly (Figure 1, Figure 2 and Figure 3). There is every virtue in rapidly scaling up vaccination, particularly in countries already suffering substantial incidence (Figure 3). Importantly, vaccination impact does not increase linearly with vaccine coverage; gains from vaccination would be proportionally higher if vaccine coverage exceeds 50% (Figure 3), stressing the importance of reaching high vaccine coverage. Easing of restrictions concurrently with vaccination can undermine many benefits of vaccination, as more people are likely to become infected before they are vaccinated. Easing of restrictions needs to be slow and gradual, tailored to the epidemiologic situation in each country (Figure S10). For instance, with its ongoing high incidence, easing of restrictions is not warranted in the US, while vaccination is scaled up.

With 95% efficacy, COVID-19 vaccination is very cost-effective, as fewer than three vaccinations are needed to avert one infection, and this effectiveness can be optimized further with more rapid scale-up (Figure 4 and Figure S8). The impact of vaccination in averting disease or death is two-fold higher for a vaccine that prevents infection compared to a vaccine that only prevents disease (Figure 1 and Figure 2 versus Figures S4 and S5). This is because preventing infection not only prevents disease directly but also reduces infection circulation, thus also indirectly reducing disease. Moreover, twice as many vaccinations are needed to avert one disease or death outcome for a vaccine that prevents only disease compared to one that prevents infection (Figure 5 and Figure S9).

This study has some limitations. Model estimations are contingent on the validity and the generalizability of input data. While we used available evidence for SARS-CoV-2 natural history and epidemiology, our understanding of its epidemiology is still evolving. All age groups were assumed equally susceptible to infection, but evidence suggests some biological differences in susceptibility [42,43,44,45,46,47,48,49]. The exact extent of exposure to the infection in both the US and China is unknown but plays an important role in vaccine impact. From an epidemiological perspective, we assumed that 20% of the US population and a negligible percentage of the Chinese population have been already infected, but vaccine impact can be quite different if such assumptions prove unrealistic. Vaccinated persons were assumed to be immediately protected once vaccinated, but in reality, vaccine protection develops over the course of a month following inoculation [6,7]. Two parameters remain unknown despite being critical to the longer-term impact of vaccination: durations of vaccine protection and natural immunity. If both prove to be relatively brief, the impact of the vaccine will be diminished, and it may be necessary to periodically re-immunize or to develop additional vaccines that protect against other circulating variants of this virus. Analysis was conducted at the national level, but operational differences at the subregional level may yield sub-regional differences in vaccination impact.

## 5. Conclusions

COVID-19 vaccination can have an immense impact on averting infection and/or disease. It can substantially flatten and delay future epidemic waves (if not prevent them altogether) and will be highly cost-effective, given the small number of vaccinations needed to avert one infection or one disease outcome. However, the impact of vaccination is likely to vary among countries, reflecting an underlying “epidemiological inequity”, as the epidemic phase in those countries also varies. Nations that will benefit most from vaccination are those where waiting time before vaccination is much shorter than time to infection, that is, countries currently at low incidence, such as China. For countries at high incidence, the impact may prove far less than current expectations, despite the vaccine’s 95% efficacy, if vaccination is scaled up slowly and/or if restrictions are eased prematurely. For countries such as the US, there is every virtue in scaling up vaccination rapidly, reaching high vaccine coverage, and delaying any easing of restrictions until viral incidence reaches low levels.

## Data Availability

All data are available in the manuscript and its Supplementary Materials. Code programmed in MATLAB can be found at: https://github.com/MouniaM/Mounia_Vacc_covid19.git (accessed on 2 February 2021).

## Supplementary Materials

The following are available online at https://www.mdpi.com/2076-393X/9/3/223/s1, Text S1A: Model Structure, Figure S1: Schematic diagram describing the SARS-CoV-2 transmission dynamics in presence of a vaccine that reduces susceptibility to infection, Table S1: Definitions of population variables and symbols used in the model, Text S1B: Model adjustment for a vaccine that reduces only severe and critical disease, Figure S2: Schematic diagram describing the SARS-CoV-2 transmission dynamics in presence of a vaccine that reduces severe and critical disease, but does not prevent infection, Figure S3: Distribution of waning of natural infection and vaccine immunity, Text S1C: Parameter values, Table S2: Model assumptions in terms of parameter values, Figure S4: Vaccine coverage scale-up, Figure S5: Impact of SARS-CoV-2 vaccination on number of (A) new infections, (B) new severe disease cases, (C) new critical disease cases, and (D) new deaths in the United States, Figure S6: Impact of SARS-CoV-2 vaccination on number of (A) new infections, (B) new severe disease cases, (C) new critical disease cases, and (D) new deaths in China, Figure S7: Impact of vaccine scale-up duration and vaccine coverage on number of averted severe and critical disease cases for a vaccine that protects only against disease, Figure S8: Impact of vaccine scale-up duration on number of vaccinations needed to avert one infection (A), one severe disease case (B), one critical disease case (C), and one death (D), in China, Figure S9: Comparison of the impact of a vaccine acting against infection (*VE_S_* efficacy) versus a vaccine acting only against disease (*VE_P_* efficacy) in China, Figure S10: Impact of the duration of easing of the social and physical distancing restrictions on number of averted severe and critical disease cases.
