# Supplementary material for: Epidemiological Differences in the Impact of COVID-19 Vaccination in the United States and China
Source: Vaccines (Basel). 2021 Mar 5;9(3):223. doi: 10.3390/vaccines9030223 (PMC8002114; doi:10.3390/vaccines9030223)
Supplement: Supplementary file 1 [file vaccines-09-00223-s001.pdf]

Supplementary Material

Epidemiological differences in the impact of COVID-19 vaccination in the United States and China

Monia Makhoul [1,2,3], Hiam Chemaitelly [1,2], Houssein H. Ayoub [4], Shaheen Seedat [1,2,3], and Laith J. Abu-Raddad [1,2.3*]

[1]Infectious Disease Epidemiology Group, Weill Cornell Medicine-Qatar, Cornell University, Qatar Foundation - Education City, Doha, Qatar

[2]World Health Organization Collaborating Centre for Disease Epidemiology Analytics on HIV/AIDS, Sexually Transmitted Infections, and Viral Hepatitis, Weill Cornell Medicine–Qatar, Cornell University, Qatar Foundation – Education City, Doha, Qatar

[3]Department of Population Health Sciences, Weill Cornell Medicine, Cornell University, New York City, New York, USA

[4]Department of Mathematics, Statistics, and Physics, Qatar University, Doha, Qatar

[*]To whom correspondence should be addressed: Email: lja2002@qatar-med.cornell.edu

Table of Contents

Text S1A. Model Structure **3**

Figure S1. Schematic diagram describing the SARS-CoV-2 transmission dynamics in presence of a vaccine that reduces susceptibility to infection **4**

Table S1. Definitions of population variables and symbols used in the model **6**

Text S1B. Model adjustment for a vaccine that reduces only severe and critical disease **8**

Figure S2. Schematic diagram describing the SARS-CoV-2 transmission dynamics in presence of a vaccine that reduces severe and critical disease, but does not prevent infection **9**

Figure S3. Distribution of waning of natural infection and vaccine immunity **10**

Text S1C. Parameter values **11**

Table S2. Model assumptions in terms of parameter values **11**

Figure S4. Vaccine coverage scale-up **14**

Figure S5. Impact of SARS-CoV-2 vaccination on number of A) new infections, B) new severe disease cases, C) new critical disease cases, and D) new deaths in the United States **15**

Figure S6. Impact of SARS-CoV-2 vaccination on number of A) new infections, B) new severe disease cases, C) new critical disease cases, and D) new deaths in China **16**

Figure S7. Impact of vaccine scale-up duration and vaccine coverage on number of averted severe and critical disease cases for a vaccine that protects only against disease **17**

Figure S8. Impact of vaccine scale-up duration on number of vaccinations needed to avert one infection (A), one severe disease case (B), one critical disease case (C), and one death (D), in China **18**

Figure S9. Comparison of the impact of a vaccine acting against infection (VE_S efficacy) versus a vaccine acting only against disease (VE_P efficacy) in China **19**

Figure S10: Impact of the duration of easing of the social and physical distancing restrictions on number of averted severe and critical disease cases **20**

Text S1. SARS-CoV-2 vaccine mathematical model

A. Model structure

We extended a recently-developed age-structured deterministic compartmental model [1-5] to describe the impact of vaccination on the severe acute respiratory syndrome coronavirus 2 (SARS-CoV-2) transmission dynamics and progression of the resulting disease, Coronavirus Disease 2019 (COVID-2019), in a given population. The model stratifies the unvaccinated and vaccinated populations into compartments according to age group (0-9, 10-19, 20-29,…, ≥80 years), infection status (uninfected, infected), infection stage (mild, severe, critical), disease stage (severe, critical), and compartments for the gamma distribution (Γ-distribution) describing the waning of natural and vaccine immunity.

Transmission and disease progression dynamics in the vaccinated and unvaccinated cohorts are described in the model using age-specific sets of nonlinear ordinary differential equations, where each age group a ($a = 1, 2, ...9$) refers to a 10-year age band (0-9,10-19,..70-79) apart from the last group including all those aged ≥0 years. The model is illustrated in Figure S1.

Figure S1. Schematic diagram describing the SARS-CoV-2 transmission dynamics model in presence of a vaccine that reduces susceptibility to infection.

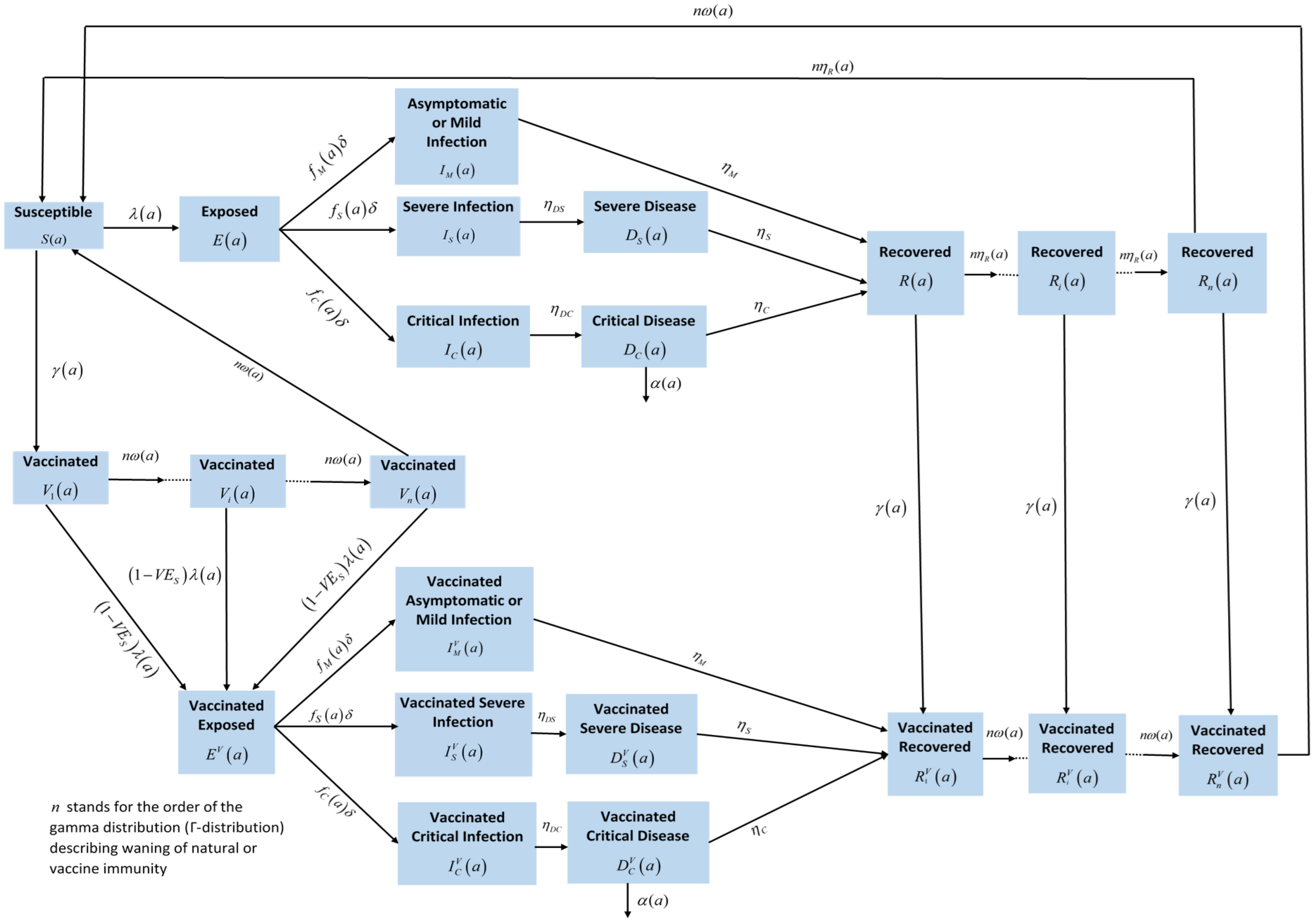

*Infection severity was per World Health Organization infection severity classification [6].

Unvaccinated population:

$$\frac{dS(a)}{dt}=\xi(a-1)S(a-1)-\left(\lambda(a)+\mu+\xi(a)+\gamma(a)\right)S(a)+n\omega(a)\left(V_n(a)+R_n^V(a)\right)+n\eta_R R_n(a)$$
$$\frac{dE(a)}{dt}=\xi(a-1)E(a-1)+\lambda(a)S(a)-\left(\delta+\mu+\xi(a)\right)E(a)$$
$$\frac{dI_M(a)}{dt}=\xi(a-1)I_M(a-1)+f_M(a)\delta E(a)-\left(\eta_M+\mu+\xi(a)\right)I_M(a)$$
$$\frac{dI_S(a)}{dt}=\xi(a-1)I_S(a-1)+f_S(a)\delta E(a)-\left(\eta_{DS}+\mu+\xi(a)\right)I_S(a)$$
$$\frac{dI_C(a)}{dt}=\xi(a-1)I_C(a-1)+f_C(a)\delta E(a)-\left(\eta_{DC}+\mu+\xi(a)\right)I_C(a)$$
$$\frac{dD_S(a)}{dt}=\xi(a-1)D_S(a-1)+\eta_{DS}I_S(a)-\left(\eta_S+\mu+\xi(a)\right)D_S(a)$$
$$\frac{dD_C(a)}{dt}=\xi(a-1)D_C(a-1)+\eta_{DC}I_C(a)-\left(\eta_C+\mu+\xi(a)+\alpha(a)\right)D_C(a)$$
$$\frac{dR_1(a)}{dt}=\xi(a-1)R_1(a-1)+\eta_M I_M(a)+\eta_S D_S(a)+\eta_C D_C(a)-\left(\mu+\xi(a)+\gamma(a)+n\eta_R\right)R_1(a)$$

For k=2,..n

$$\frac{dR_k(a)}{dt}=\xi(a-1)R_k(a-1)+n\eta_R R_{k-1}(a)-\left(\mu+\xi(a)+\gamma(a)+n\eta_R\right)R_k(a)$$

Vaccinated populations aged 10+ years:

$$\frac{dV_1(a)}{dt}=\xi(a-1)V_1(a-1)+\gamma(a)S(a)-\left((1-VE_S)\lambda(a)+\mu+\xi(a)+n\omega(a)\right)V_1(a)$$

For k=2,..n

$$\frac{dV_k(a)}{dt}=\xi(a-1)V_k(a-1)+n\omega(a)V_{k-1}(a)-\left((1-VE_S)\lambda(a)+\mu+\xi(a)+n\omega(a)\right)V_k(a)$$
$$\frac{dE^V(a)}{dt}=\xi(a-1)E^V(a-1)+(1-VE_S)\lambda(a)\left(\sum_{k=1}^{n}V_k(a)\right)-\left(\delta+\mu+\xi(a)\right)E^V(a)$$
$$\frac{dI_M^V(a)}{dt}=\xi(a-1)I_M^V(a-1)+f_M(a)\delta E^V(a)-\left(\eta_M+\mu+\xi(a)\right)I_M^V(a)$$
$$\frac{dI_S^V(a)}{dt}=\xi(a-1)I_S^V(a-1)+f_S(a)\delta E^V(a)-\left(\eta_{DS}+\mu+\xi(a)\right)I_S^V(a)$$
$$\frac{dI_C^V(a)}{dt}=\xi(a-1)I_C^V(a-1)+f_C(a)\delta E^V(a)-\left(\eta_{DC}+\mu+\xi(a)\right)I_C^V(a)$$
$$\frac{dD_S^V(a)}{dt}=\xi(a-1)D_S^V(a-1)+\eta_{DS}I_S^V(a)-\left(\eta_S+\mu+\xi(a)\right)D_S^V(a)$$

$$\frac{dD_C^V(a)}{dt} = \xi(a-1)D_C^V(a-1) + \eta_{DC} I_C^V(a) - \left(\eta_C + \mu + \xi(a) + \alpha(a)\right) D_C^V(a)$$

$$\frac{dR_1^V(a)}{dt} = \xi(a-1)R_1^V(a-1) + \gamma(a)R_1(a) + \eta_M I_M^V(a) + \eta_S D_S^V(a) + \eta_C D_C^V(a) - \left(\mu + \xi(a) + n\omega(a)\right) R_1^V(a)$$

For k=2,..n

$$\frac{dR_k^V(a)}{dt} = \xi(a-1)R_k^V(a-1) + \gamma(a)R_k(a) + n\omega(a)R_{k-1}^V(a) - \left(\mu + \xi(a) + n\omega(a)\right) R_k^V(a)$$

Where n stands for the order of the gamma distribution (Γ-distribution) describing waning of natural or vaccine immunity.

The definitions of population variables and symbols used in the equations are in Table S1.

Table S1. Definitions of population variables and symbols used in the model.

Symbol	Definition
**Transmission dynamics parameters**	
$S(a)$	Unvaccinated susceptible population
$E(a)$	Unvaccinated latently infected population
$I_M(a)$	Unvaccinated population with asymptomatic or mild infection
$I_S(a)$	Unvaccinated population with severe infection
$I_C(a)$	Unvaccinated population with critical infection
$D_S(a)$	Unvaccinated population with severe disease
$D_C(a)$	Unvaccinated population with critical disease
$R_i(a)$	i^{th} compartment to generate the gamma distribution for the waning of natural immunity among the unvaccinated recovered population
$V_i(a)$	i^{th} compartment to generate the gamma distribution for the waning of vaccine immunity among the vaccinated susceptible population
$E^V(a)$	Vaccinated latently infected population
$I_M^V(a)$	Vaccinated population with asymptomatic or mild infection
$I_S^V(a)$	Vaccinated population with severe infection
$I_C^V(a)$	Vaccinated population with critical infection
$D_S^V(a)$	Vaccinated population with severe disease
$D_C^V(a)$	Vaccinated population with critical disease
$R_i^V(a)$	i^{th} compartment to generate the gamma distribution for the waning of natural immunity among the vaccinated recovered population
N	Total population size
n_{age}	Number of age groups
n	Order of the gamma distribution (Γ-distribution) describing waning of natural or vaccine immunity
$\xi(a)$	Transition rate from one age group to the next age group
β	Overall infectious contact rate

$1/\delta$	Duration of latent infection
$1/\eta_M$	Duration of asymptomatic or mild infection
$1/\eta_{DS}$	Duration of severe infection infectiousness before isolation and/or hospitalization
$1/\eta_S$	Duration of severe disease following onset of severe disease
$1/\eta_{DC}$	Duration of critical infection infectiousness before isolation and/or hospitalization
$1/\eta_C$	Duration of critical disease following onset of critical disease
$1/\eta_R$	Duration of natural immunity
$1/\mu$	Natural death rate
$\alpha(a)$	Mortality rate in each age group
$f_M(a)$	Proportion of infections that will progress to be mild or asymptomatic infections
$f_S(a)$	Proportion of infections that will progress to be severe infections
$f_C(a)$	Proportion of infections that will progress to be critical infections
**Key vaccine product characteristics**	
VE_s	Vaccine efficacy in reducing susceptibility
VE_P	Vaccine efficacy in reducing severe and critical disease
$1/\omega$	Duration of vaccine protection

The force of infection (hazard rate of infection) experienced by the unvaccinated susceptible populations $S(a)$ is given by

$$\lambda(a) = \beta \sum_{a'=1}^{n_{age}} H_{a,a'} \frac{I_M(a') + I_S(a') + I_C(a') + I_M^V(a') + I_S^V(a') + I_C^V(a')}{\left[\begin{array}{l} S(a') + E(a') + I_M(a') + I_S(a') + I_C(a') + D_S(a') + D_C(a') + \sum_{i=1}^{n} R_i(a') + \\ \sum_{i=1}^{n} V_i(a') + E^V(a') + I_M^V(a') + I_S^V(a') + I_C^V(a') + D_S^V(a') + D_C^V(a') + \sum_{i=1}^{n} R_i^V(a') \end{array}\right]},$$

while that experienced by the vaccinated susceptible populations $V(a)$ is given by

$$\lambda^V(a) = (1 - VE_S)\lambda(a)$$

where β is the overall infectious contact rate. The mixing among the different age groups is dictated by the mixing matrix $H_{a,a''}$. This matrix provides the probability that an individual in the a age group will mix with an individual in the a' age group (regardless of vaccination status).

The mixing matrix is given by

$$H_{a,a'} = e_{Age}\delta_{a,a'} + (1-e_{Age})\frac{\begin{bmatrix} S(a')+E(a')+I_M(a')+I_S(a')+I_C(a')+D_S(a')+D_C(a')+\sum_{i=1}^{n} R_i^V(a')+ \\ \sum_{i=1}^{n} V_i(a')+E^V(a')+I_M^V(a')+I_S^V(a')+I_C^V(a')+D_S^V(a')+D_C^V(a')+\sum_{i=1}^{n} R_i^V(a') \end{bmatrix}}{\sum_{a=1}^{n_{age}}\begin{bmatrix} S(a)+E(a)+I_M(a)+I_S(a)+I_C(a)+D_S(a)+D_C(a)+\sum_{i=1}^{n} R_i^V(a)+ \\ \sum_{i=1}^{n} V_i(a)+E^V(a)+I_M^V(a)+I_S^V(a)+I_C^V(a)+D_S^V(a)+D_C^V(a)+\sum_{i=1}^{n} R_i^V(a) \end{bmatrix}}$$

Here, $\delta_{a,a'}$ is the identity matrix. $e_{Age} \in [0,1]$ measures the degree of assortativeness in the mixing. At the extreme $e_{Age} = 0$, the mixing is fully proportional. Meanwhile, at the other extreme, $e_{Age} = 1$, the mixing is fully assortative, that is individuals mix only with members in their own age group.

B. Model adjustment for a vaccine that reduces only severe and critical disease

The above model was adjusted to accommodate for a vaccine that has no effect on infection, but that reduces both severe and critical disease with an efficacy VE_P. A schematic diagram of the adjusted model is provided in Figure S2.

Figure S2. Schematic diagram describing the SARS-CoV-2 transmission dynamics model in presence of a vaccine that reduces severe and critical disease, but does not prevent infection.

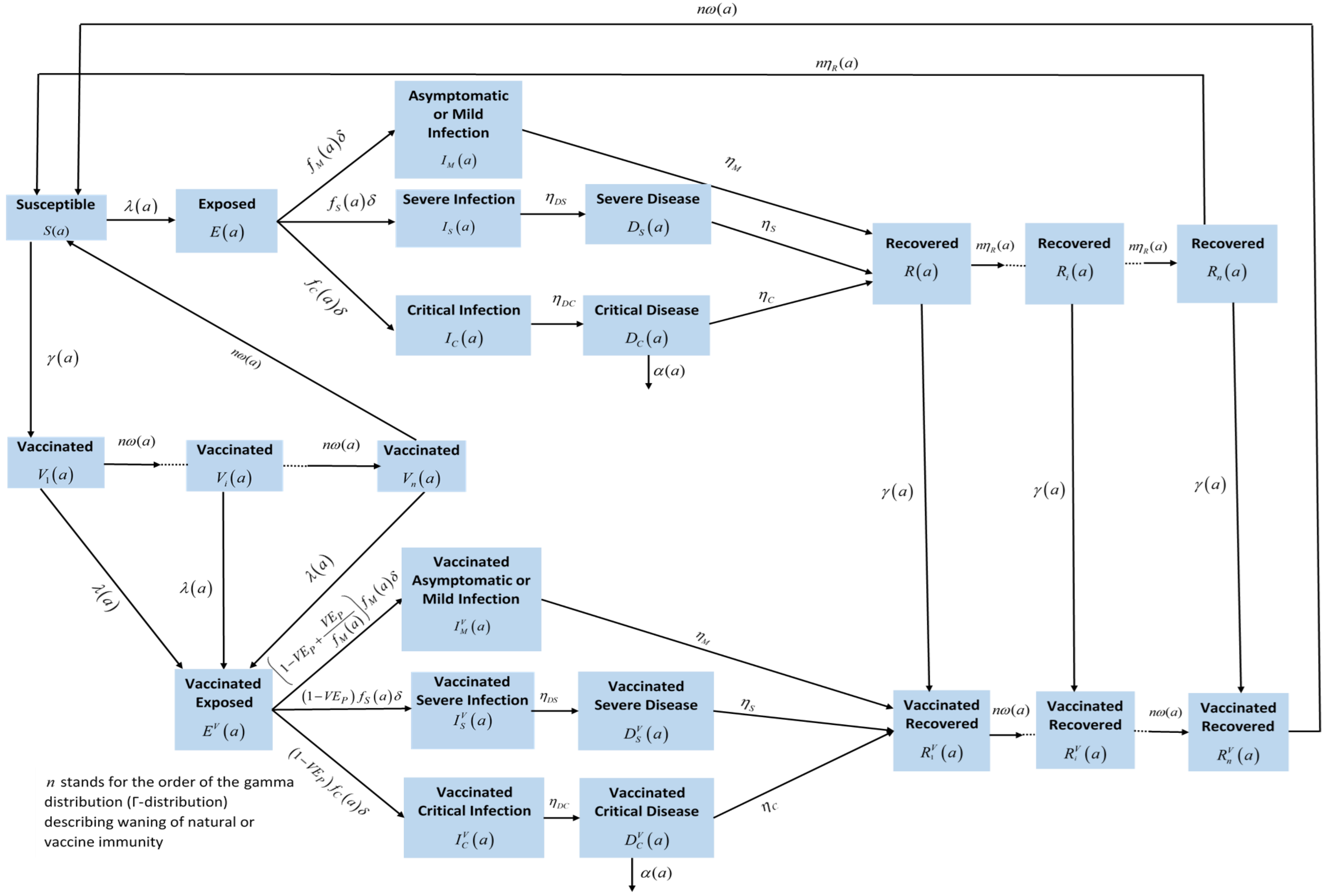

*Infection severity was per World Health Organization infection severity classification [6].

Figure S3. Time course of waning natural infection and vaccine immunity. Waning of natural infection and vaccine immunity was parametrized using a gamma distribution with order $n = 15$. That is, most people lose their immunity after about one year and only a small minority lose it much sooner or much later than one year.

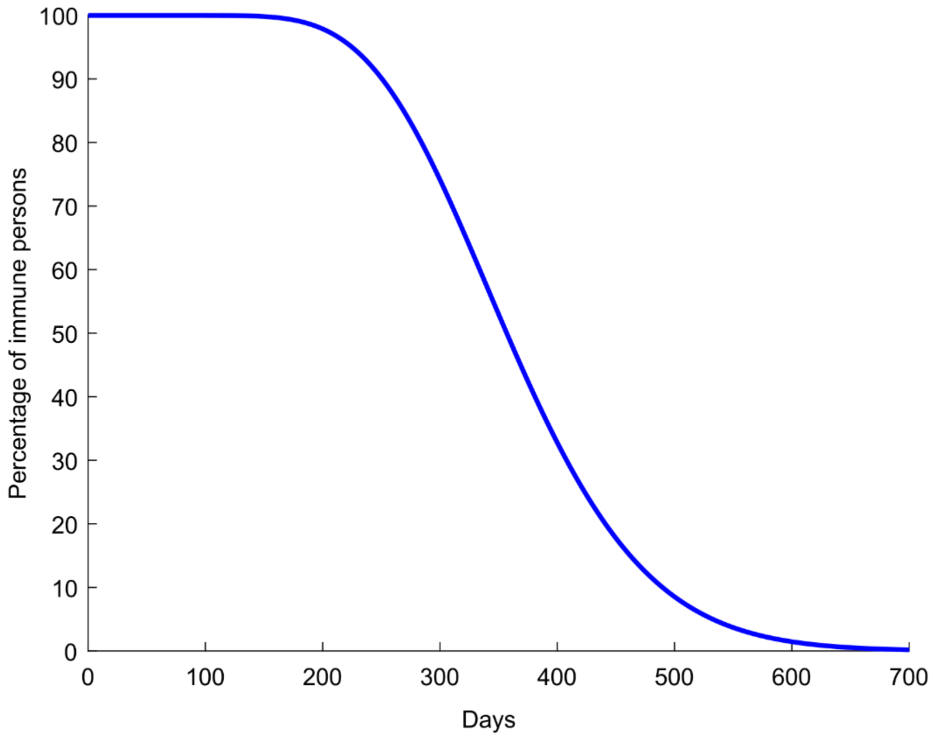

C. Parameter values

The input parameters of the model were chosen based on current empirical data for SARS-CoV-2 natural history and epidemiology. The parameter values are listed in Table S2.

Table S2. Model assumptions in terms of parameter values

Parameter	Symbol	Value	Justification
Duration of latent infection	$1/\delta$	3.69 days	Based on existing estimate [7] and based on a median incubation period of 5.1 days [8] adjusted by observed viral load among infected persons [9] and reported transmission before onset of symptoms [10]
Duration of infectiousness	$1/\eta_M$;<br>$1/\eta_{DS}$;<br>$1/\eta_{DC}$	3.48 days	Based on existing estimate [7] and based on observed time to recovery among persons with mild infection [7, 11] and observed viral load in infected persons [9, 10, 12]
Duration of severe disease following onset of severe disease	$1/\eta_S$	28 days	Observed duration from onset of severe disease to recovery [11]
Duration of hospitalization for critical infection	$1/\eta_C$	42 days	Observed duration from onset of critical disease to recovery [11]
Life expectancy in US<br>Life expectancy in China	$1/\mu$<br>$1/\mu$	79.10 years<br>76.47 years	United Nations World Population Prospects database [13]
Proportion of infections that will progress to be mild or asymptomatic infections	$f_M(a)$	Determined from $f_M(a)+f_S(a)+f_C(a)=1$	Observed proportion of infections that eventually develop mild or asymptomatic in France [11, 14, 15]
Proportion of infections that will progress to be infections that require hospitalization in acute care beds	$f_S(a)$		The distribution and age dependence of asymptomatic/mild, severe, or critical infections was based on the modeled SARS-CoV-2 epidemic in France [16]
Age 0-19 years	$RRS1\times f_S$	$RRS1=0.1$	Model-estimated relative risk of severe infection based on the SARS-CoV-2 epidemic in France [16]
Age 20-29 years	$RRS2\times f_S$	$RRS2=0.5$	Model-estimated relative risk of severe infection based on the SARS-CoV-2 epidemic in France [16]
Age 30-39 years	f_S	Reference category (f_S =0.01)	Model-estimated based on fitting the SARS-CoV-2 epidemic in France [16]
Age 40-49 years	$RRS3\times f_S$	$RRS3=1.2$	Model-estimated relative risk of severe infection based on the SARS-CoV-2 epidemic in France [16]
Age 50-59 years	$RRS4\times f_S$	$RRS4=2.3$	Model-estimated relative risk of severe infection based on the SARS-CoV-2 epidemic in France [16]

Age 60-69 years	$RRS5\times f_S$	$RRS5=4.5$	Model-estimated relative risk of severe infection based on the SARS-CoV-2 epidemic in France [16]
Age 70-79 years	$RRS6\times f_S$	$RRS6=7.8$	Model-estimated relative risk of severe infection based on the SARS-CoV-2 epidemic in France [16]
Age 80+ years	$RRS7\times f_S$	$RRS7=27.6$	Model-estimated relative risk of severe infection based on the SARS-CoV-2 epidemic in France [16]
Proportion of infections that will progress to be critical infections	$f_C(a)$		The distribution and age dependence of asymptomatic/mild, severe, or critical infections was based on the modeled SARS-CoV-2 epidemic in France [16]
Age 0-19 years	$RRC1\times f_C$	$RRC1=0.21$	Model-estimated relative risk of critical infection based on the SARS-CoV-2 epidemic in France [16]
Age 20-29 years	$RRC2\times f_C$	$RRC2=0.33$	Model-estimated relative risk of critical infection based on the SARS-CoV-2 epidemic in France [16]
Age 30-39 years	f_C	Reference category $f_C=0.0002$	Model-estimated based on fitting the SARS-CoV-2 epidemic in France [16]
Age 40-49 years	$RRC3\times f_C$	$RRC3=1.83$	Model-estimated relative risk of critical infection based on the SARS-CoV-2 epidemic in France [16]
Age 50-59 years	$RRC4\times f_C$	$RRC4=4.67$	Model-estimated relative risk of critical infection based on the SARS-CoV-2 epidemic in France [16]
Age 50-59 years	$RRC4\times f_C$	$RRC4=4.67$	Model-estimated relative risk of critical infection based on the SARS-CoV-2 epidemic in France [16]
Age 60-69 years	$RRC5\times f_C$	$RRC5=10.58$	Model-estimated relative risk of critical infection based on the SARS-CoV-2 epidemic in France [16]
Age 70-79 years	$RRC6\times f_C$	$RRC6=13.61$	Model-estimated relative risk of critical infection based on the SARS-CoV-2 epidemic in France [16]
	$RRC7\times f_C$	$RRC7=8.67$	Model-estimated relative risk of critical infection based on the SARS-CoV-2 epidemic in France [16]
Disease mortality rate in each age group	$\alpha(a)$		The distribution and age dependence of COVID-19 mortality was based on the modeled SARS-CoV-2 epidemic in France [16]
Age 0-19 years	$RRD1\times\alpha$	$RRD1=0.10$	Model-estimated relative risk of death based on the SARS-CoV-2 epidemic in France [16]
Age 20-29 years	$RRD2\times\alpha$	$RRD2=0.40$	Model-estimated relative risk of death based on the SARS-CoV-2 epidemic in France [16]
Age 30-39 years	α	Reference category (α =0.0006)	Model-estimated based on fitting the SARS-CoV-2 epidemic in France [16]
Age 40-49 years	$RRD3\times\alpha$	$RRD3=3.00$	Model-estimated relative risk of death based on the SARS-CoV-2 epidemic in France [16]

Age 50-59 years	$RRD4 \times \alpha$	$RRD4 = 10.00$	Model-estimated relative risk of death based on the SARS-CoV-2 epidemic in France [16]
Age 60-69 years	$RRD5 \times \alpha$	$RRD5 = 45.00$	Model-estimated relative risk of death based on the SARS-CoV-2 epidemic in France [16]
Age 70-79 years	$RRD6 \times \alpha$	$RRD6 = 120.00$	Model-estimated relative risk of death based on the SARS-CoV-2 epidemic in France [16]
	$RRD7 \times \alpha$	$RRD7 = 505.00$	Model-estimated relative risk of death based on the SARS-CoV-2 epidemic in France [16]
Overall infectious contact rate	β	0.33 contacts per day	Chosen to yield the desired value of $R_0 = 1.2$ in the US
		0.28 contacts per day	Chosen to yield the desired value of $R_0 = 1.0$ in China

Figure S4. Vaccine coverage scale-up. Proportion of the cumulative number of vaccinated people in A) the United States and B) China over 2021.

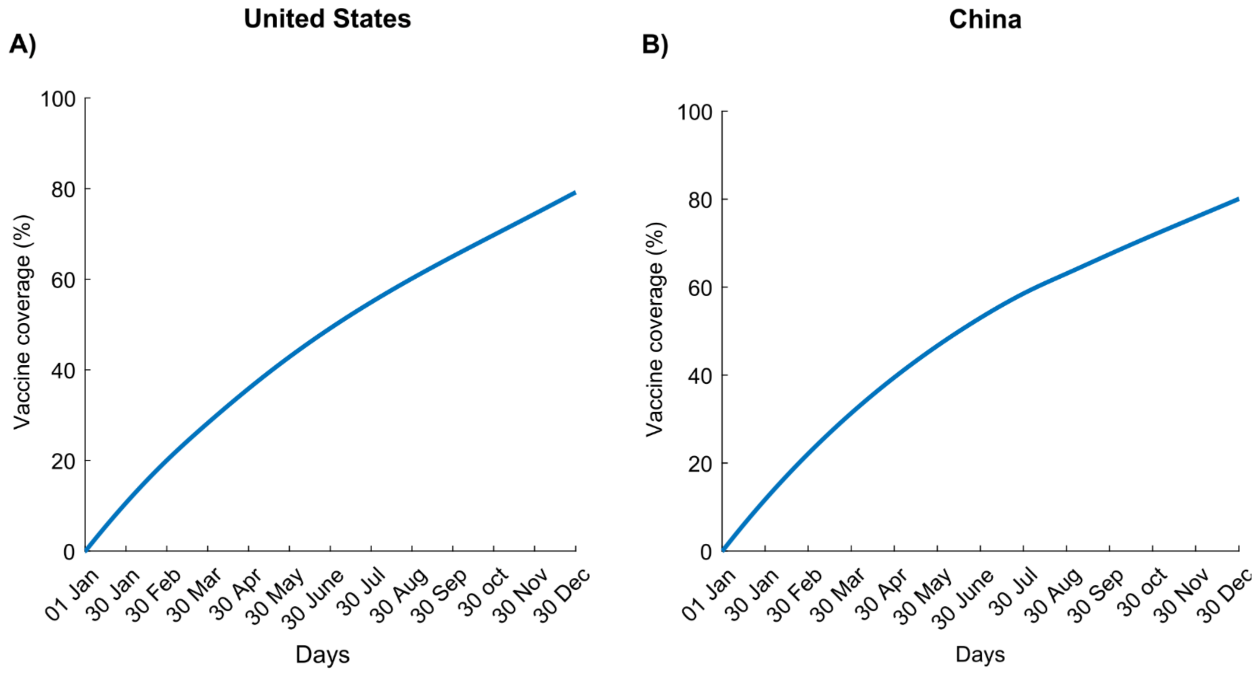

Figure S5. Impact of SARS-CoV-2 vaccination on numbers of A) new infections, B) new severe disease cases, C) new critical disease cases, and D) new deaths in the United States. The vaccine is assumed to have no efficacy against infection, but an efficacy of 95% against severe and critical disease. It is introduced on January 1, 2021, when the cumulative proportion of the population infected is 20%. Vaccine coverage is scaled up to reach 80% by December 31, 2021. The duration of both vaccine protection and natural immunity is one year. This scenario assumes an R_0 of 1.2 on January 1, 2021, which increases with gradual easing of restrictions to reach 4.0 after six months.

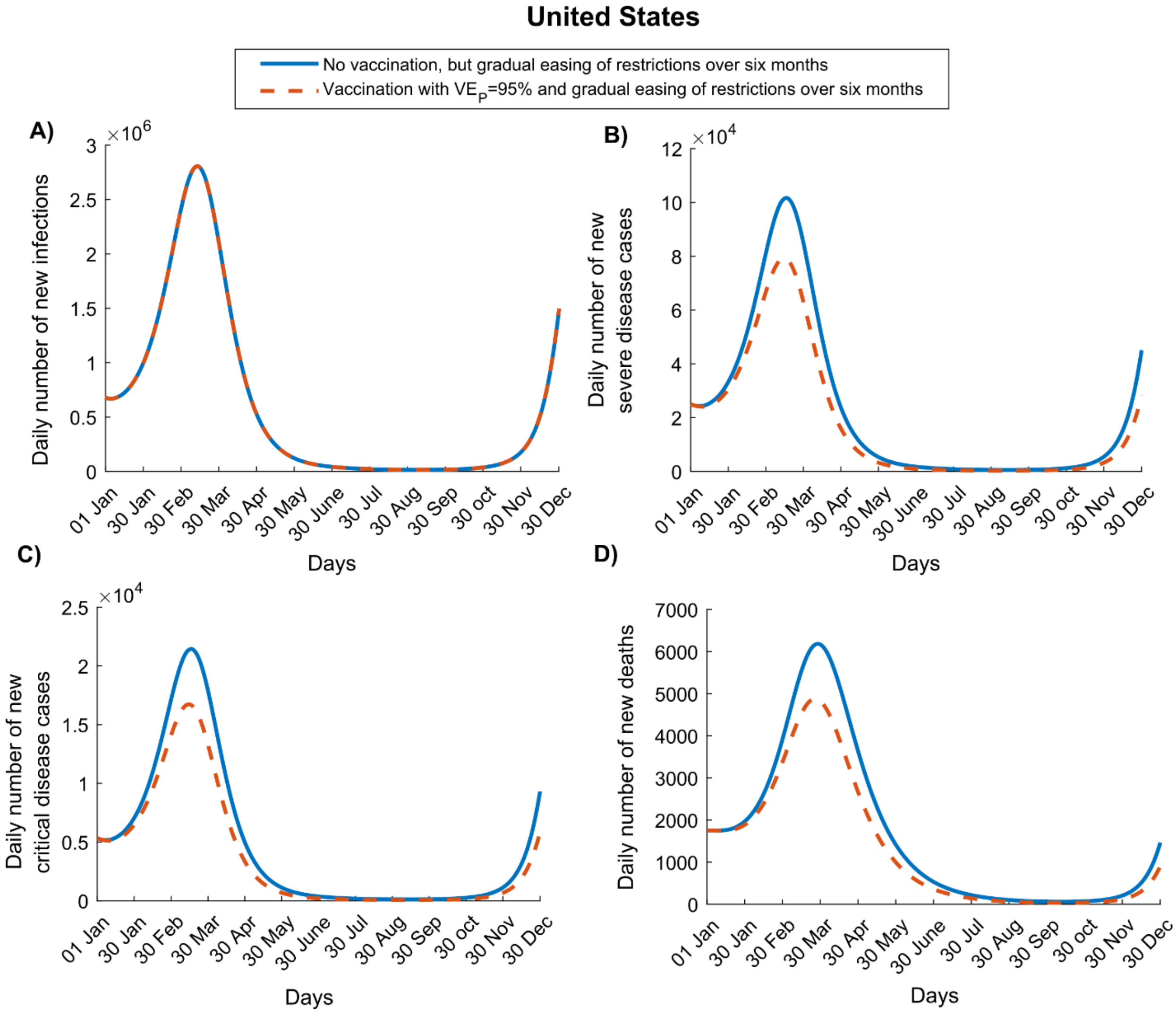

Figure S6. **Impact of SARS-CoV-2 vaccination on numbers of A) new infections, B) new severe disease cases, C) new critical disease cases, and D) new deaths in China**. The vaccine is assumed to have no efficacy against infection, but an efficacy of 95% against severe and critical disease and is introduced on January 1, 2021. Vaccine coverage is scaled up to reach 80% by December 31, 2021. The duration of both vaccine protection and natural immunity is one year. This scenario assumes an R_0 of 1.0 on January 1, 2021, which increases with the gradual easing of restrictions to reach 4.0 after six months.

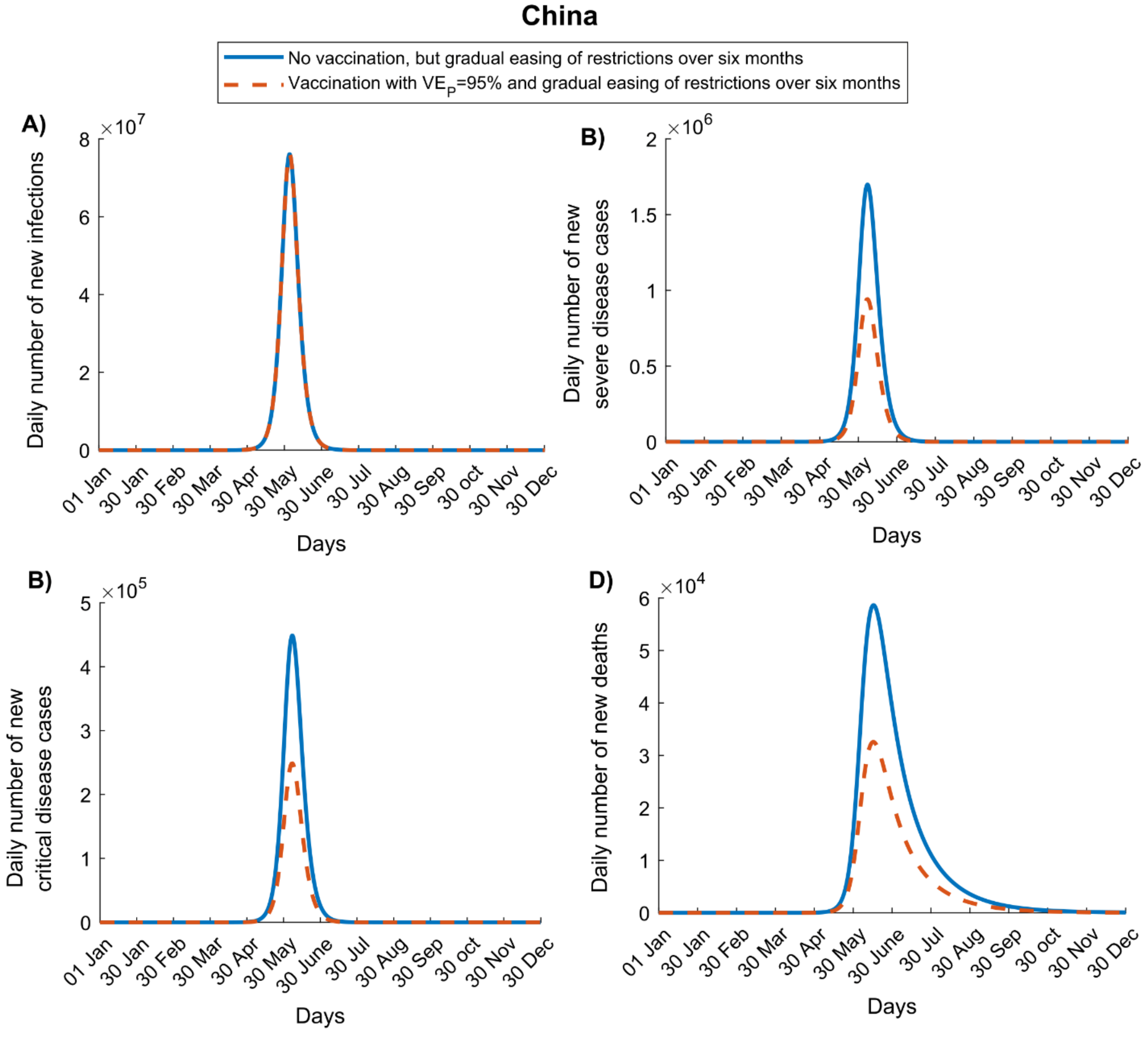

Figure S7. Impact of vaccine scale-up duration and vaccine coverage on numbers of averted severe and critical disease cases for a vaccine that protects only against disease. Cumulative numbers of averted severe and critical disease cases in A) the United States and C) China at different vaccination scale-up intervals to reach 80% coverage. Cumulative numbers of averted severe and critical disease cases in B) the United States and D) China at varying levels of vaccine coverage. The vaccine is assumed to have no efficacy against infection, but an efficacy of 95% against severe and critical disease and is introduced on January 1, 2021, when the cumulative proportion of the population infected is 20% in the United States and 0% in China. The duration of both vaccine protection and natural immunity is one year. This scenario assumes a gradual easing of restrictions within 6 months.

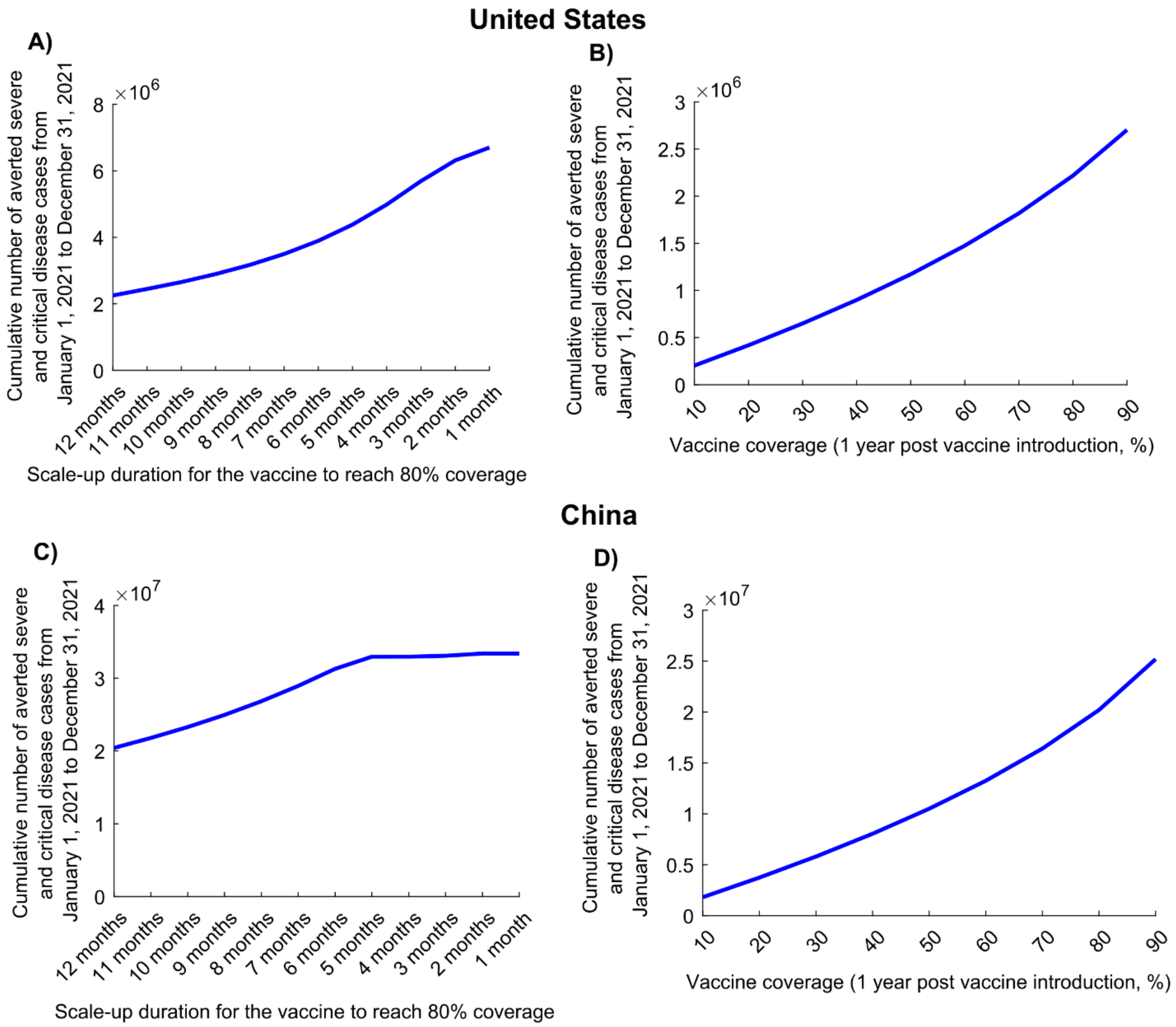

Figure S8. Impact of vaccine scale-up duration on the number of vaccinations needed to avert one infection (A), one severe disease case (B), one critical disease case (C), and one death (D) in China. The vaccine is assumed to have an efficacy of 95% against infection and is introduced on January 1, 2021, when the cumulative proportion of the population infected is 0%. The duration of both vaccine protection and natural immunity is one year. This scenario assumes a gradual easing of restrictions within 6 months.

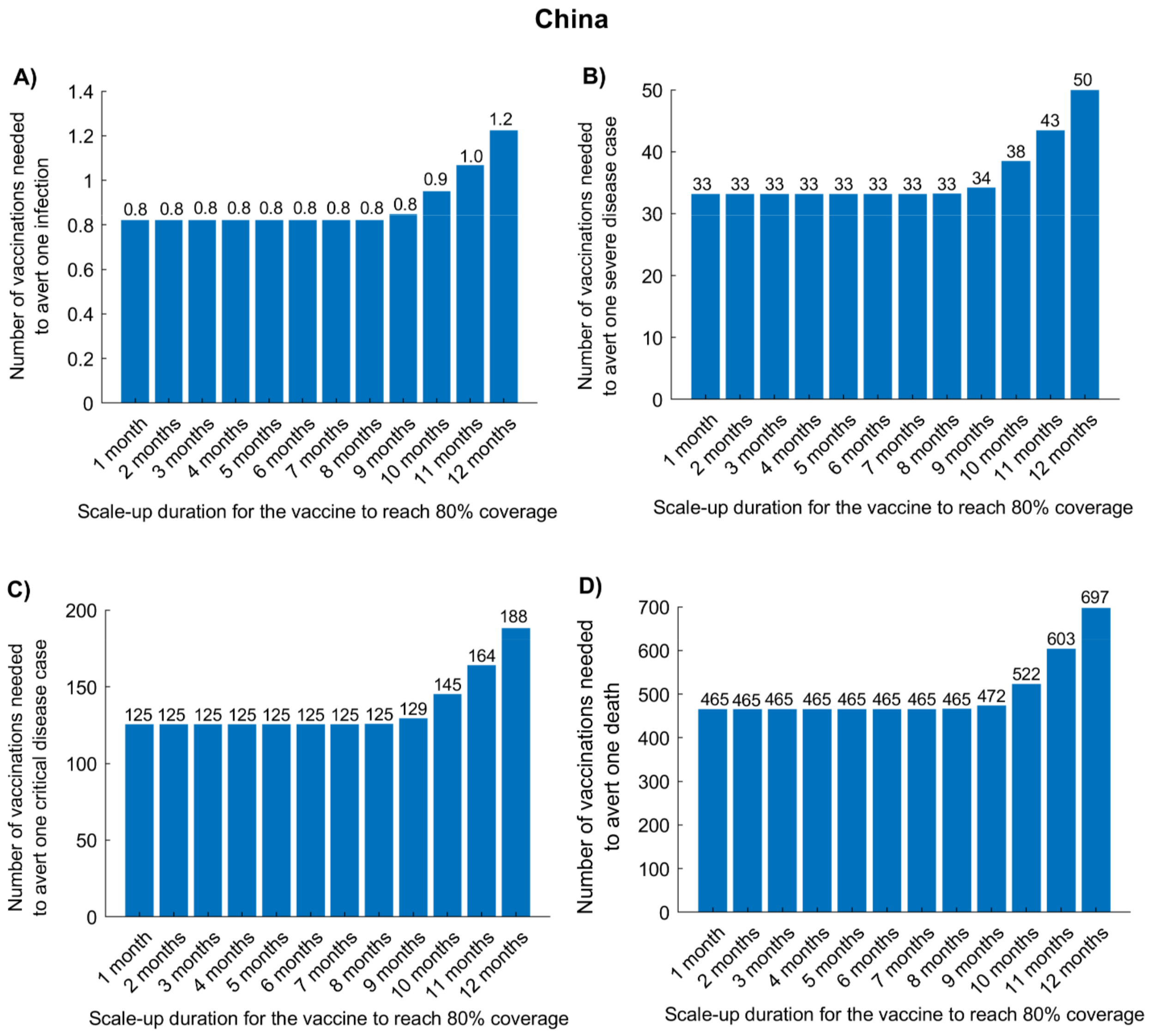

Figure S9. Comparison of the impact of a vaccine acting against infection (*VE_S* efficacy) versus a vaccine acting only against disease (*VE_P* efficacy) in China. The number of vaccinations needed to avert one severe disease case (A), one critical disease case (B), and one death (C), for a vaccine with $VE_S = 95\%$ versus a vaccine with $VE_P = 95\%$. The vaccine is introduced on January 1, 2021, when the cumulative proportion of the population infected is 0%. The duration of both vaccine protection and natural immunity is one year. This scenario assumes a gradual easing of restrictions within 6 months.

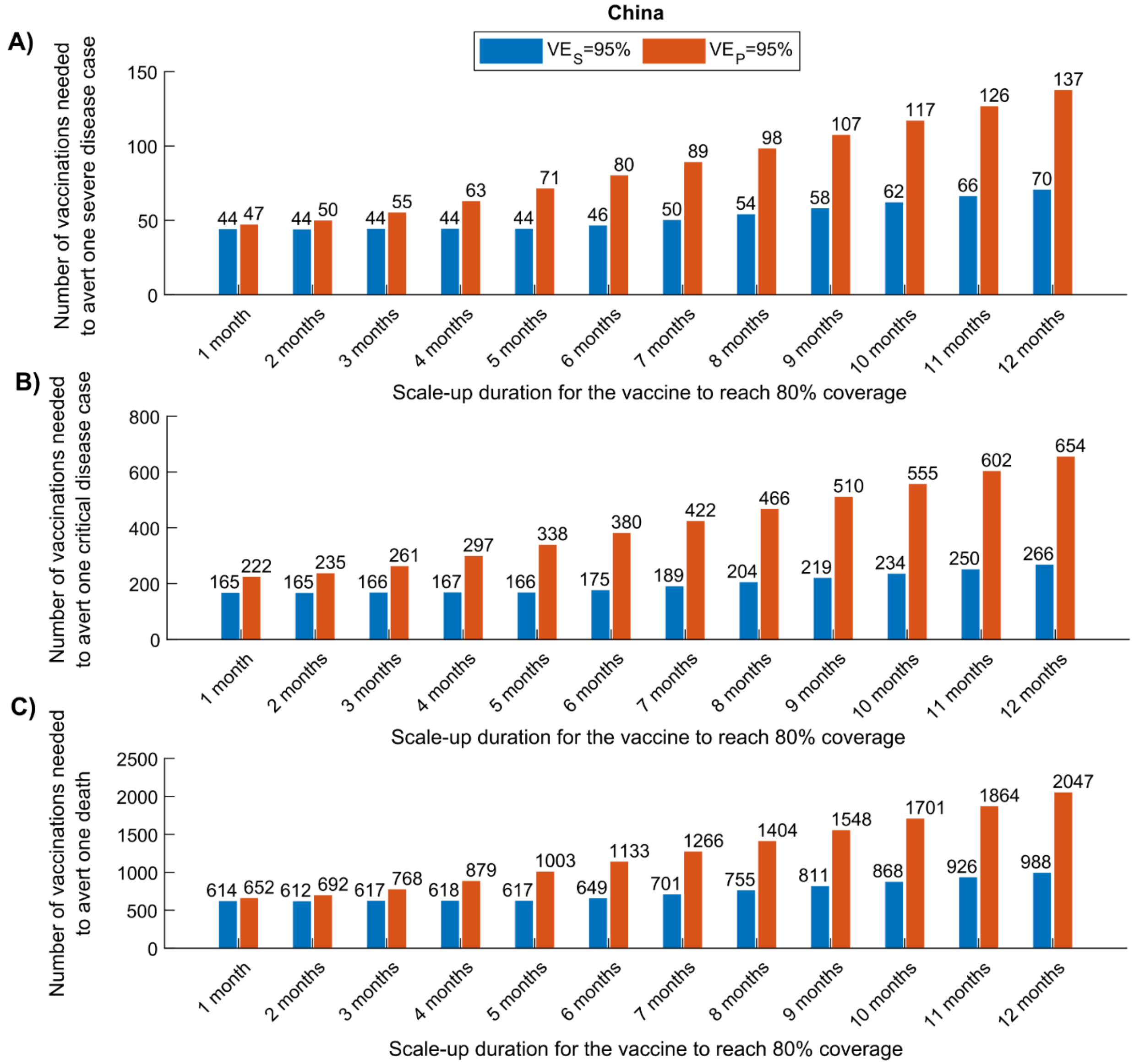

Figure S10. Impact of the duration of easing social and physical distancing restrictions on the number of averted severe and critical disease cases. The cumulative number of averted severe and critical disease cases in A) the United States and B) China at different durations of easing of restrictions. The vaccine is assumed to have an efficacy of 95% against infection and is introduced on January 1, 2021, when the cumulative proportion of the population infected is 20% in the United States and 0% in China. The duration of both vaccine protection and natural immunity is one year.

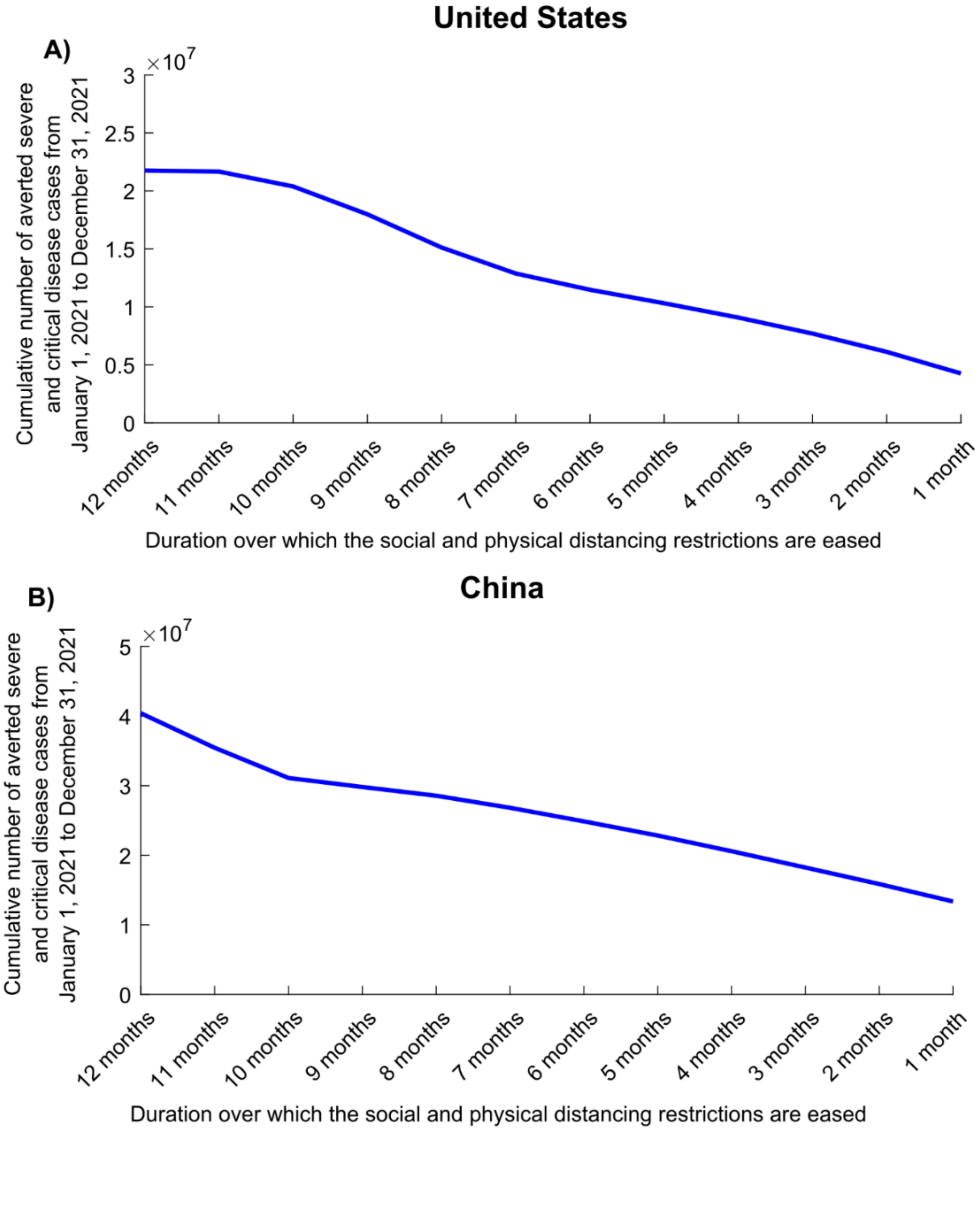

References

1 Makhoul M, Ayoub HH, Chemaitelly H, Seedat S, Mumtaz GR, Al-Omari S, et al. Epidemiological Impact of SARS-CoV-2 vaccination: mathematical modeling analyses. Vaccines. 2020;8.

2 Ayoub HH, Chemaitelly H, Seedat S, Mumtaz GR, Makhoul M, Abu-Raddad LJ. Age could be driving variable SARS-CoV-2 epidemic trajectories worldwide. PLOS ONE. 2020;15:e0237959.

3 Ayoub HH, Chemaitelly H, Mumtaz GR, Seedat S, Awad SF, Makhoul M, et al. Characterizing key attributes of COVID-19 transmission dynamics in China's original outbreak: model-based estimations. Global epidemiology. 2020;2:100042.

4 Makhoul M, Abou-Hijleh F, Seedat S, Mumtaz GR, Chemaitelly H, Ayoub H, et al. Analyzing inherent biases in SARS-CoV-2 PCR and serological epidemiologic metrics. BMC- ID.Under review.

5 Seedat S, Chemaitelly H, Ayoub H, Makhoul M, Mumtaz GR, Kanaani ZA, et al. SARS-CoV-2 infection hospitalization, severity, criticality, and fatality rates. medRxiv. 2020:2020.11.29.20240416.

6 World Health Organization. Clinical management of COVID-19. Available from: https://www.who.int/publications-detail/clinical-management-of-covid-19. Accessed on: May 31st 2020. 2020.

7 Li R, Pei S, Chen B, Song Y, Zhang T, Yang W, et al. Substantial undocumented infection facilitates the rapid dissemination of novel coronavirus (SARS-CoV2). Science. 2020;368:489-93.

8 Lauer SA, Grantz KH, Bi Q, Jones FK, Zheng Q, Meredith HR, et al. The Incubation Period of Coronavirus Disease 2019 (COVID-19) From Publicly Reported Confirmed Cases: Estimation and Application. Ann Intern Med. 2020;172:577-82.

9 Zou L, Ruan F, Huang M, Liang L, Huang H, Hong Z, et al. SARS-CoV-2 Viral Load in Upper Respiratory Specimens of Infected Patients. N Engl J Med. 2020;382:1177-9.

10 Rothe C, Schunk M, Sothmann P, Bretzel G, Froeschl G, Wallrauch C, et al. Transmission of 2019-nCoV Infection from an Asymptomatic Contact in Germany. N Engl J Med. 2020;382:970-1.

11 World Health Organization. Report of the WHO-China Joint Mission on Coronavirus Disease 2019 (COVID-19). Available from :https://www.who.int/docs/default-source/coronaviruse/who-china-joint-mission-on-covid-19-final-report.pdf. Accessed on March 10, 2020. 2020.

12 He X, Lau EHY, Wu P, Deng X, Wang J, Hao X, et al. Temporal dynamics in viral shedding and transmissibility of COVID-19. Nat Med. 2020;26:672-5.

13 United Nations Department of Economic and Social Affairs Population Dynamics. The 2019 Revision of World Population Prospects. Available from https://population.un.org/wpp/. Accessed on March 1st, 2020. 2020.

14 Guan WJ, Ni ZY, Hu Y, Liang WH, Ou CQ, He JX, et al. Clinical Characteristics of Coronavirus Disease 2019 in China. N Engl J Med. 2020.

15 Huang C, Wang Y, Li X, Ren L, Zhao J, Hu Y, et al. Clinical features of patients infected with 2019 novel coronavirus in Wuhan, China. Lancet. 2020;395:497-506.

16 Salje H, Tran Kiem C, Lefrancq N, Courtejoie N, Bosetti P, Paireau J, et al. Estimating the burden of SARS-CoV-2 in France. Science. 2020:eabc3517.
